# Longitudinal Assessment of Health-Related Quality of Life in Older Adults Receiving Rehabilitative Care in a Japanese Geriatric Health Service Facility

**DOI:** 10.7759/cureus.101301

**Published:** 2026-01-11

**Authors:** Nobuichiro Tamura, Seigo Urushidani, Tetsunori Ikegami

**Affiliations:** 1 Emergency and Critical Care Center, Kurashiki Central Hospital, Kurashiki, JPN

**Keywords:** ascot, community dwelling elderly, geriatric health service facility, hrqol, longitudinal study

## Abstract

Introduction: This exploratory study examined longitudinal trends in health-related quality of life (HRQoL) among older adults receiving short-term inpatient rehabilitation at a Japanese geriatric health service facility (ROKEN).

Methods: A prospective observational study was conducted at one ROKEN facility in Kurashiki City. Participants admitted for approximately three months with planned discharge home were assessed using the SF-12 (PCS, MCS, RCS) and ASCOT SCT4 at three time points: pre-admission (T1), one month after admission (T2), and one month post-discharge (T3). Friedman and Wilcoxon signed-rank tests were applied.

Results: Of 49 participants enrolled, 24 completed all assessments. Mental health (MCS) showed a modest improvement from T1 to T3 (p=0.04), while role-related and social domains (RCS and ASCOT) declined after discharge. Satisfaction with personal care, food, and accommodation remained high, whereas control over daily life and social participation decreased.

Conclusions: These preliminary findings suggest that inpatient rehabilitation may support mental health, but social and role-related aspects tend to worsen after discharge. High attrition and small sample size limit generalizability, underscoring the need for further research and enhanced post-discharge support.

## Introduction

Japan is experiencing unprecedented acceleration in population aging, which has led to a sharp increase in the proportion of older adults. This demographic shift has placed growing pressure on the healthcare and social care systems [1]. The growing demand for long-term care and medical services highlights the urgent need for sustainable policies and systems. In response, the Japanese government introduced a long-term care insurance system to support older adults living at home after being discharged from hospitals or other facilities [2]. This system aims to maintain independence and quality of life while reducing the pressure on healthcare resources. Simultaneously, advances in often costly medical technologies have shifted policy discussions toward value-based, patient-centered care, with greater emphasis on health-related quality of life (HRQoL) [3].

Within this framework, Japan has promoted comprehensive community-based care through multidisciplinary collaborations among physicians, nurses, rehabilitation specialists, and dietitians [2]. Geriatric health-service facilities, also known as ROKEN, play a central role. ROKEN provides intensive inpatient rehabilitation, typically for three months, to improve activities of daily living (ADL) and promote independence. After discharge, patients typically return home for approximately three months before possible readmission if further rehabilitation is needed. This cyclical model of alternating between home- and facility-based rehabilitation is unique to Japan and is designed to support community living through short-term, targeted interventions. Recently, the use of patient-reported outcomes (PROs) has been recognized as essential to patient-centered care [4]. However, empirical assessments of HRQoL among users remain limited.

HRQoL is influenced by living environment and lifestyle [5], yet existing studies have largely focused on either community-dwelling older adults [5,6] or institutionalized populations [7,8], without tracking the same individuals across care transitions. To our knowledge, no prior research has examined longitudinal changes in HRQoL throughout the unique ROKEN care cycle in Japan, which alternates between home and institutional rehabilitation. This study addresses this critical gap by evaluating HRQoL at three time points within this cycle, providing novel insights into care transition effects in a super-aged society.

Accordingly, the main aim of this study was to longitudinally assess HRQoL at three time points: prior to ROKEN admission (T1), one month after admission (T2), and one month following discharge (T3), to clarify patterns of change during transitions between home and facility-based care.

HRQoL encompasses three key dimensions: physical, mental, and social roles. In Japanese culture, social roles are considered to contribute substantially to overall HRQoL. For example, while the summary scores of the SF-36 in Western countries typically include physical and mental components, the Japanese version additionally incorporates social role functioning [9]. Therefore, the present study aimed to clarify longitudinal changes in HRQoL, with a particular focus on the social role component among these three dimensions. We hypothesized that HRQoL would progressively improve over time across the three assessment points (T1, T2, and T3).

## Materials and methods

Study design

This prospective observational study was conducted at a single ROKEN institution in Kurashiki City, Okayama Prefecture, Japan. As of 2021, Kurashiki City has an estimated population of approximately 1.876 million residents, of whom 575,000 are aged 65 years or older, corresponding to a senior population ratio of 30.6%, which closely approximates the national average for Japan of 30.3% in the same year [1]. The inclusion period spanned between November 1, 2022, and March 31, 2024. Ethical approval for the study protocol was obtained from the Institutional Review Board of Kurashiki Central Hospital (No.3914). Prior to admission to the ROKEN facility, trained staff members conducted home visits to provide a detailed explanation of the study objectives and procedures and obtain written informed consent from all participants.

Study settings

The primary goal of this study was to examine the trajectory of HRQoL in community-dwelling older adults within the same cohort as their living environment transitioned from home to institutional care. To achieve this objective, three summary scores derived from the SF-12 and the Adult Social Care Outcomes Toolkit (ASCOT) SCT4 were selected as primary outcome measures. Data were collected at three distinct time points. The first assessment (T1) was conducted during the pre-admission home visit, during which the participants completed self-administered questionnaires covering demographic information, SF-12, and ASCOT SCT4. The second assessment (T2) took place one month after admission, at which time the facility staff again requested participants to complete the same self-administered questionnaires. The third assessment (T3) was conducted one month after discharge; questionnaires were mailed to the participants’ homes, and follow-up phone calls were made if the completed forms were not returned, ensuring comprehensive data collection.

The SF-12 is available in a standard version, using a one-month recall period, and an acute version, with a one-week recall. This study used the standard version to match the intervals between living environment transitions and minimize recall bias. The second and third assessments (T2 and T3) were conducted about one month after each transition to align with the instrument’s recall period.

Population

Participants were residents admitted to the ROKEN facility between November 2022 and March 2024, expected to be discharged to their own homes after approximately three months. Because HRQoL is influenced by the post-discharge living environment [10], individuals planned for discharge to long-term care facilities other than their own homes were excluded. The participants also needed sufficient cognitive capacity to complete self-reported questionnaires. Those unable to complete the questionnaires because of cognitive impairment, visual deficits, or other impairments during the pre-admission assessments were excluded. Decisional capacity to complete the HRQoL questionnaires was determined by ROKEN staff during the pre-admission home visit. Ideally, MMSE would precede questionnaire administration; however, the pre-admission visit is time-limited and such testing was not feasible in routine practice. Instead, trained staff-who possess extensive caregiving experience and, in many cases, prior familiarity with the prospective participant-made a pragmatic judgment based on direct observation and interaction. Additionally, participants who developed complications requiring hospital transfer or whose functional status did not improve, resulting in discharge to a nursing home instead of home, were excluded from the outcome analyses.

Outcome measures

Prior to admission, ROKEN staff collected data on participants’ age, sex, annual income, educational background, living situation (living alone or with others), marital status, smoking status, certified care level, number of previous ROKEN admissions, primary disease requiring care, duration of caregiving, Hospital Anxiety and Depression Scale (HADS) scores [11], and Mini-Mental State Examination (MMSE) scores [12].

Functional Independence Measure (FIM) [13] assessments were performed by rehabilitation therapists trained in standardized scoring procedures. Annual income was categorized into quartiles based on national statistics: ≤1.5 million yen, 1.51-2.52 million yen, 2.53-4.24 million yen, and ≥4.25 million yen [1].

The SF-12 generates scores across eight health domains: physical functioning, physical role, bodily pain, general health, vitality, social functioning, emotional role, and mental health, while producing three summary scores in the Japanese version: the Physical Component Summary (PCS), Mental Component Summary (MCS), and Role/Social Component Summary (RCS). The SF-12 has been validated in Japanese populations with established national normative values [9,14]. Scores were standardized with a mean of 50 and standard deviation of 5, enabling cross-population comparisons.

The ASCOT SCT4 assesses social care-related quality of life (SCRQoL) across eight domains: control over daily life, personal cleanliness and comfort, food and drink, personal safety, social participation, occupation, accommodation cleanliness and comfort, and dignity. Responses were rated on four levels, ranging from an ideal state to high-level needs. Using a preference-weighted scoring algorithm, the total scores ranged from −0.17 (worst QoL) to 1 (best QoL), with 0 representing a state equivalent to death. The Japanese preference weights were developed to ensure culturally relevant scoring [15]. While total scores are typically the primary analytic focus [16], exploratory analyses were conducted on individual domain responses to elucidate patterns of change [17].

SF-12 scoring followed the developer’s standard norm-based procedures for the Japanese version; forms with missing SF-12 items at a given time point were excluded from summary score computation at that time point. ASCOT SCT4 was scored using the Japanese preference-weighted algorithm; questionnaires with missing ASCOT SCT4 items were excluded at that time point. We did not impute missing item responses; instead, we conducted complete-case analyses per time point and compared baseline characteristics between included and excluded participants to assess attrition bias.

Sample size

No formal sample size calculation was performed because this study was exploratory and aimed to generate preliminary insights into HRQoL changes across care transitions. The sample size was based on the number of eligible participants admitted to the facility during the inclusion period, reflecting real-world feasibility rather than statistical power considerations.

Data analysis

Qualitative variables were expressed as counts and percentages, and continuous variables were summarized using median and interquartile ranges (IQRs). Comparisons between participants included in the final analysis and those excluded were conducted using Pearson’s chi-square test for categorical variables and the Mann-Whitney U test for continuous variables to assess potential attrition bias. We examined histograms, Q-Q plots, and Shapiro-Wilk tests for all summary scores. Because distributions were non-normal and some measures exhibited skew and ceiling effects, we summarized continuous variables using medians and interquartile ranges and applied non-parametric tests.

HRQoL outcomes (SF-12 PCS, MCS, RCS, and ASCOT SCT4) were initially compared across T1, T2, and T3 using Friedman’s test. For variables with p-values <0.05, post hoc pairwise comparisons were conducted using Wilcoxon signed-rank tests, applying the Bonferroni correction. We calculated effect sizes (Kendall’s W for Friedman tests; matched-pairs rank-biserial correlation for Wilcoxon tests) and Hodges-Lehmann estimates with 95% confidence intervals. Statistical significance was defined as p<0.05, two-sided. The analyses were performed using EZR version 1.61 [18].

## Results

Population characteristics

**Figure 1:**
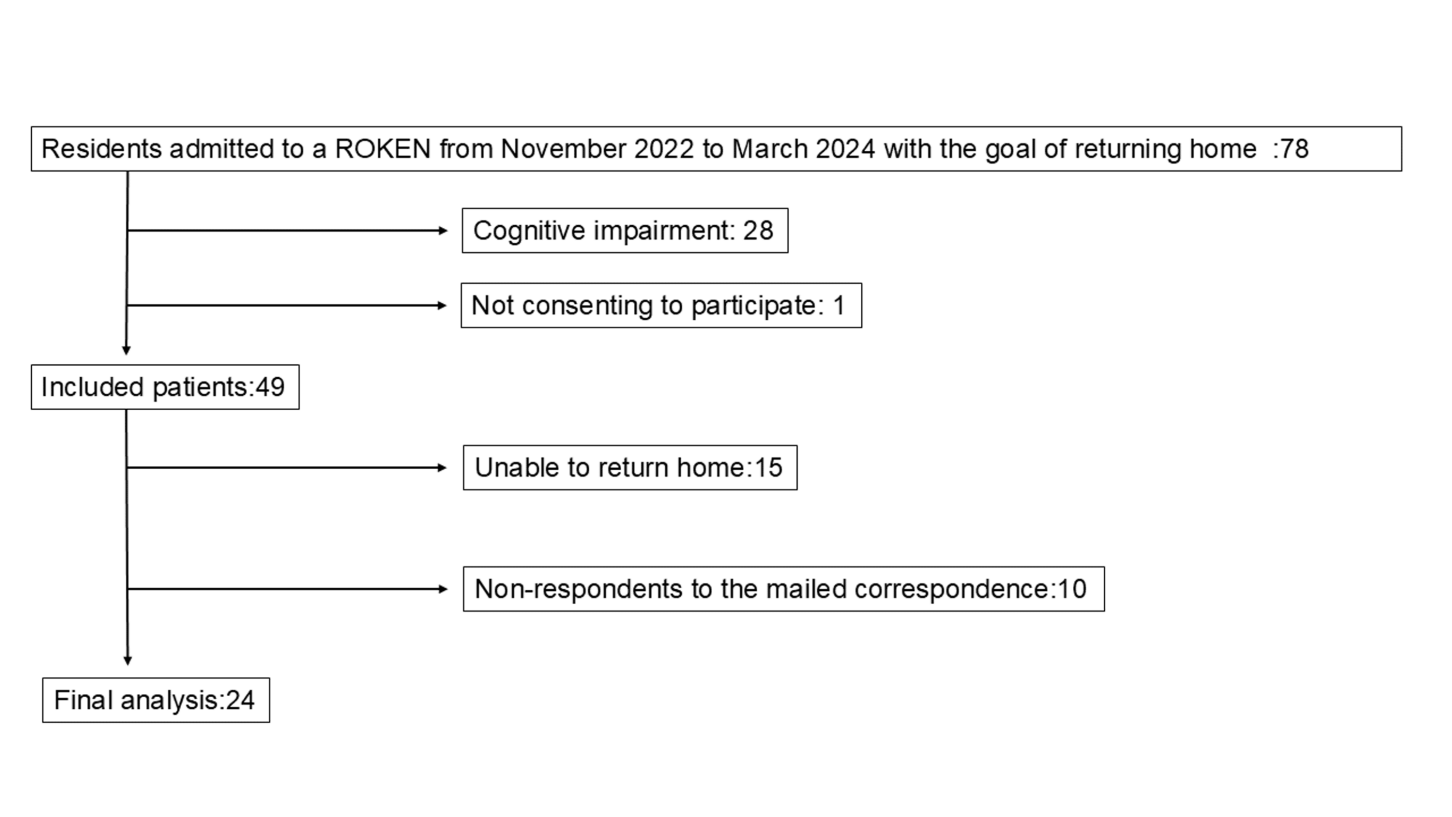
A flow diagram of participant recruitment

Between November 2022 and March 2024, 78 participants were admitted to the ROKEN facility in Kurashiki City to return home after rehabilitation. Twenty-eight patients were excluded due to cognitive impairment precluding self-completion of the questionnaires, and one declined consent, leaving 49 patients enrolled. During follow-up, 25 (51.0%) patients were excluded: 15 (30.6%) were discharged to nursing facilities as they could not return home, and 10 (20.4%) returned home but did not complete the T3 assessment. The final analysis included 24 (49.0%) participants (Figure 1).

The baseline characteristics of the 24 patients are presented in Table 1. The median age was 80.5 years, with a predominance of females (n=16, 66.7%). The most common annual income was 1.51-2.52 million yen (n=11, 45.8%), and high school graduates constituted the largest educational group (n=13, 54.2%). Five participants (20.8%) lived alone, most lived with their families, and 13 (54.2%) were widowed. Sixteen patients (66.7%) had never smoked before. Fourteen participants (58.3%) required severe care (Level 3 or higher). The median FIM score was 93.5. The median MMSE score were 22.0. The median HADS scores were 4.5 for anxiety and 9.0 for depression. The median number of prior ROKEN admissions was 4.0, and the median caregiving duration was 33.5 months. Stroke was the most common primary diagnosis (n=12, 50.0%).

**Table 1:** The characteristics of the 24 participants included in the final data analysis and the 25 participants who dropped out HADS: Hospital Anxiety and Depression Scale; FIM: Functional Independence Measure; MMSE: Mini-Mental State Examination

	Final analysis (n=24)	Drop out (n=25)	p-value
Age	80.5(IQR:72.8-86.5)	83.0(IQR:75.0-89.0)	0.38
Sex			
Male	8(33.3%)	8(32.0%)	1.00
Female	16(66.7%)	17(68.0%)	
Annual income (KJPY)			
1500>	8(33.3%)	10(40.0%)	0.45
1501-2520	11(45.8%)	7(28.0%)	
2521-4240	1(4.2%)	4(16.0%)	
>4241	4(16.7%)	4(16.0%)	
Education background			
Junior high school	8(33.3%)	9(36%)	0.28
High school	13(54.2%)	16(64%)	
Vocational School	3(12.5%)	0(0.0%)	
Living situation			
Alone	5(20.8%)	5(20.0%)	1.00
With family	19(79.2%)	20(80.0%)	
Marital status			
With spouse	8(33.3%)	12(48.0%)	0.59
Never married	3(12.5%)	2(8.0%)	
Divorced	0(0.0%)	1(4.0%)	
Widowed	13(54.2%)	10(40.0%)	
Disease			
Stroke	12(50.0%)	8(32.0%)	0.33
Fracture	2(8.3%)	6(24.0%)	
Neurological disorder	5(20.8%)	2(8.0%)	
Disuse syndrome	1(4.2%)	3(12.0%)	
Others	4(16.7%)	6(24.0%)	
Smoking status			
Never	16(66.7%)	14(56.0%)	0.18
Past	6(25.0%)	11(44.0%)	
Current	2(8.0%)	0(0.0%)	
Care level certification			
Care-need level 1	5(20.8%)	7(28.0%)	0.69
Care-need level 2	5(20.8%)	6(24.0%)	
Care-need level 3	9(37.5%)	4(16.0%)	
Care-need level 4	3(12.5%)	5(20.0%)	
Care-need level 5	2(8.3%)	3(12.0%)	
Number of previous admissions to ROKEN	4.0(IQR:2.0-5.3)	2.0(IQR:1.0-3.0)	0.01
Care-needed duration (months)	33.5(IQR:18.8-60.0)	12.0(IQR:6.0-31.0)	0.01
HADS-anxiety	4.5(IQR:2.0-8.5)	4.5(IQR:2.0-8.5)	0.39
HADS-depression	9.0(IQR:5.0-12.0)	8.0(IQR:5.0-11.0)	0.70
FIM-motor	61.5(IQR:52.5-72.3)	63.0(IQR:54.0-75.0)	0.79
FIM-cognitive	27.0(IQR:22.0-29.0)	24.0(IQR:22.0-29.0)	0.59
FIM-total	93.5(IQR:78.0-98.0)	95.0(IQR:78.0-100.0)	0.78
MMSE	22.0(IQR:21.0-24.3)	22.0(IQR:20.0-25.0)	0.95

A comparison of the final 24 participants with the 25 excluded participants revealed significant differences in prior ROKEN admissions and caregiving duration, whereas age, sex, and baseline ADL were comparable, indicating that the final cohort was broadly representative.

Outcomes

Table 2 summarizes the longitudinal changes in HRQoL measures at T1, T2, and T3. Median SF-12 PCS scores were 26.5, 31.1, and 22.2 at T1, T2, and T3, respectively. The median MCS scores were 57.9, 61.1, and 61.1. The median RCS scores were 40.8, 49.8, and 27.3. ASCOT SCT4 scores demonstrated a median of 0.61 at T1, 0.75 at T2, and 0.52 at T3.

**Table 2:** Longitudinal changes in SF-12 and ASCOT SCT4 at T1, T2, and T3 with effect sizes r_rb: Rank-biserial correlation; HL diff: Hodges–Lehmann median difference; ASCOT: Adult Social Care Outcomes Toolkit; RCS: Role/Social Component Summary SF-12 PCS had no significant overall change; pairwise effect sizes not presented For RCS, two significant pairwise contrasts are shown: † = T2→T3 , ‡ = T1→T3

	T1 (median (IQR))	T2 (median (IQR))	T3 (median (IQR ))	p-value (Friedman)	Kendall W	p-value (T1 vs T2)	p-value (T2 vs T3)	p-value (T1 vs T3)	r_rb	HL diff (95%CI)
SF-12 PCS	26.5 (17.6–35.5)	31.1 (24.8–36.8)	22.2 (16.8–31.7)	0.07	0.036	—	—	—	—	—
SF-12 MCS	57.9 (49.5–63.5)	61.1 (55.9–67.0)	61.1 (56.2–68.8)	0.03	0.049	0.06	0.79	0.04	+0.48 (T1→T3)	+4.10 (+0.30, +11.20)
SF-12 RCS	40.8 (35.8–55.0)	49.8 (40.9–56.8)	27.3 (19.0–42.8)	0.02	0.054	0.19	<0.01	0.03	−0.50†; −0.42‡	−16.80 (−26.30, −10.10)†; −12.15 (−25.90, −4.20)‡
ASCOT SCT4	0.61 (0.47–0.75)	0.75 (0.61–0.81)	0.52 (0.32–0.73)	0.03	0.047	0.17	0.02	0.13	−0.58 (T2→T3)	−0.173 (−0.284, −0.014)

Friedman tests revealed statistically significant changes over time in MCS (p=0.03), RCS (p=0.02), and ASCOT SCT4 scores (p=0.03). Post hoc Wilcoxon signed-rank tests, adjusted for multiple comparisons using the Bonferroni correction, indicated significant increases in the MCS between T1 and T3 (p=0.04). The RCS scores showed a significant decline between T2 and T3 (p<0.01) and between T1 and T3 (p=0.03). The ASCOT SCT4 scores decreased significantly from T2 to T3 (p=0.02).

Effect sizes were calculated for all tests (Kendall’s W for Friedman tests; matched-pairs rank-biserial correlation for Wilcoxon tests) along with Hodges-Lehmann estimates and 95% confidence intervals. For SF-12 PCS, Kendall’s W was 0.036, indicating a very small effect, consistent with the non-significant Friedman test (p = 0.07). For SF-12 MCS, Kendall’s W was 0.049, and the rank-biserial correlation for the significant T1-T3 comparison was +0.48, with a Hodges-Lehmann median difference of +4.10 (95% CI: +0.30 to +11.20). For SF-12 RCS, Kendall’s W was 0.054; the rank-biserial correlations were −0.50 for T2-T3 and −0.42 for T1-T3, with Hodges-Lehmann differences of −16.80 (95% CI: −26.30 to −10.10) and −12.15 (95% CI: −25.90 to −4.20), respectively. For ASCOT SCT4, Kendall’s W was 0.047, and the rank-biserial correlation for T2-T3 was −0.58, with a Hodges-Lehmann difference of −0.173 (95% CI: −0.284 to −0.014).

**Figure 2:**
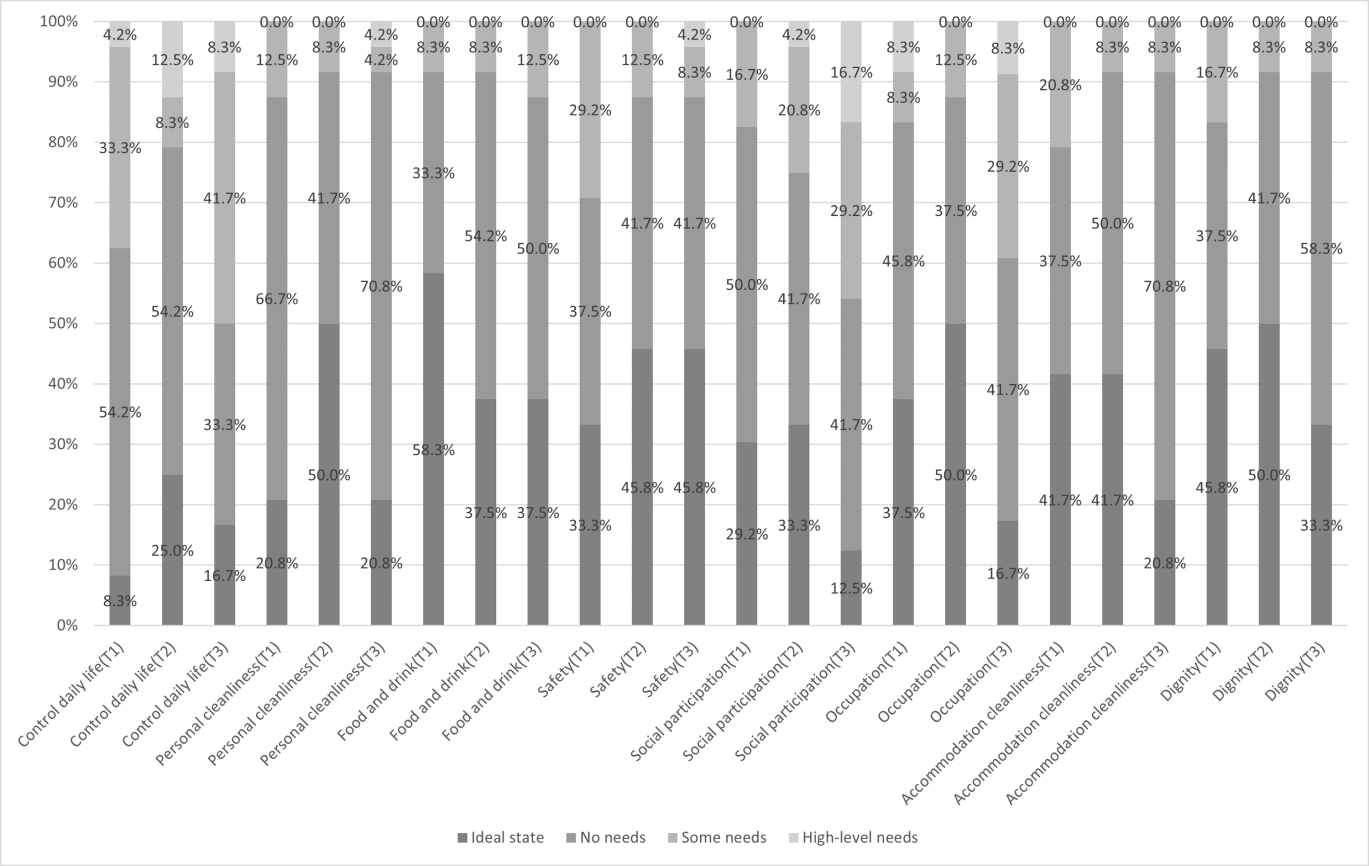
Proportion of responses for each ASCOT SCT4 questionnaire ASCOT: Adult Social Care Outcomes Toolkit

Figure 2 illustrates the domain-specific ASCOT SCT4 responses. Across all time points, Personal cleanliness, Food and drink, and Accommodation cleanliness domains maintained high levels of satisfaction, with approximately 80-90% of the participants reporting an ideal state or no needs. Conversely, domains reflecting social engagement, Control over daily life, Social participation, and Occupation, demonstrated a notable decline at T3, with only 50-60% reporting positive outcomes. These findings suggest that, although basic care needs were adequately met, engagement in social and meaningful activities declined after returning home.

## Discussion

This study is the first prospective longitudinal investigation of HRQoL among older adults transitioning between home and institutional care in a super-aged society, focusing on ROKEN facilities where admissions and discharges follow a cyclical three-month pattern. Using the SF-12 and ASCOT SCT4 as complementary measures, we observed significant improvements in the SF-12 MCS during ROKEN admission, but notable declines in the SF-12 RCS and ASCOT SCT4 after discharge. Among these, the most pronounced change was in the RCS, indicating a perceived reduction in social functioning following discharge. Effect size analysis further supported these findings: the improvement in MCS from T1 to T3 demonstrated a moderate effect (r = 0.48), whereas the decline in RCS after discharge showed a large negative effect (r = −0.50), underscoring the clinical relevance of these changes. Similarly, ASCOT SCT4 exhibited a moderate decrease (r = −0.58) between T2 and T3, suggesting a substantial deterioration in social care-related quality of life post-discharge.

Social roles are known to be closely associated with HRQoL. For example, Kishikawa et al. reported significant positive correlations between social roles and the “Psychological” and “Social Relationships” domains of the WHOQOL-26 among elderly day care users in Japan [19]. Similar associations have been observed in Germany, where social roles were linked to life satisfaction among community-dwelling older adults [20]. Based on these findings, the decline in RCS scores after discharge may reflect reduced opportunities for social engagement. During ROKEN stays, patients often participate in rehabilitation activities and interact with staff and peers, which may foster a sense of social role fulfillment. After returning home, these opportunities could diminish, potentially contributing to lower perceived social functioning.

According to the International Classification of Functioning, Disability, and Health, rehabilitation goals should extend beyond improving body function and activities to include participation in social life [21]. At ROKEN, rehabilitation staff aim to prepare patients for social participation post-discharge, for example, through training in household tasks such as dishwashing. However, once at home, family members may limit older adults’ activities due to concerns about falls or overprotection, which could reduce opportunities for independence and social engagement. Cultural norms in some Asian contexts may further discourage active use of functional abilities, potentially exacerbating this trend [22]. While these factors offer plausible explanations for the observed decline, the observational design of this study does not allow for definitive conclusions regarding causality. Future research should explore these mechanisms more rigorously and examine interventions that involve both facility-based rehabilitation and family education to support sustained social participation after discharge.

In this study, median ASCOT SCT4 scores changed from 0.61 (IQR: 0.47-0.75) at T1 to 0.75 (IQR: 0.61-0.81) at T2, before declining to 0.52 (IQR: 0.32-0.73) at T3, with T3 significantly lower than T2. The ASCOT SCT4 was designed to evaluate HRQoL from a social care perspective, focusing on the needs of older adults and individuals with disabilities rather than medical outcomes. Although large-scale intervention studies using the ASCOT SCT4 have been reported in the UK and Australia, research remains limited in Japan [23]. Whitehead et al. examined a home-based intervention (“Home Care Re-ablement Service”) for older adults with functional limitations, finding ASCOT SCT4 improvements of 0.04-0.07 points from baseline (0.77) over six months [24]. By contrast, our study demonstrated a significant decline between T2 and T3. A possible explanation for this is the difference in baseline HRQoL; participants in Whitehead’s study had higher initial scores and sufficient functioning to remain at home, whereas our participants required ROKEN admission and had lower baseline scores. Prior longitudinal research has suggested that a higher baseline HRQoL predicts better maintenance or improvement over time [25,26]. The decline observed in this study likely reflects these baseline differences.

Although the ASCOT SCT4 is usually analyzed using the total score, we conducted exploratory domain-level analyses. Regarding Personal cleanliness, Food and drink, Accommodation cleanliness, and Dignity, over 80% of the participants consistently reported an ideal state or no needs at T1-T3. In contrast, positive ratings for Control over daily life, Social participation, and Occupation declined to 50-60% at T3. Similarly, the RCS of SF-12 significantly decreased. Because the ASCOT SCT4 domains, such as Social participation and Occupation and the RCS, both assess social roles and engagement, the consistency of these findings supports their reliability.

Participants transitioned from living at home (T1) to a ROKEN facility (T2), and then back home (T3). While ROKEN provided balanced meals, a safe environment, and clean clothing and bedding, high satisfaction with Personal cleanliness, Food and drink, and Accommodation cleanliness was maintained at all time points. This indicates that participants may not perceive the environmental differences between facility- and home-based care as strongly as providers do. Similarly, a UK study of reablement services found that Occupation, Social contact, and Control over daily life were the domains most likely to change, consistent with our results [17]. These domains reflect subjective aspects of daily life that are not well captured by ADL-based measures such as the FIM.

Thus, healthcare and social care providers should assess not only physical function but also personal perceptions. Incorporating patient-reported outcomes, such as ASCOT SCT4 and SF-12, may provide a more comprehensive evaluation of QoL in older adults.

Strengths and limitations

Strengths

This study was uniquely positioned to capture longitudinal HRQoL changes across a care trajectory distinctive to Japan, involving cyclic transitions between home and ROKEN. A prospective design within a single cohort allows within-subject analysis to minimize confounding due to inter-individual variability. To our knowledge, no prior studies have evaluated this care pathway longitudinally using both the SF-12 and ASCOT SCT4.

Limitations

This study has several important limitations. First, it was conducted at a single ROKEN facility with a relatively small sample size, which restricts external validity. Although the aging rate in Kurashiki approximates the national average, caution is warranted when generalizing these findings to other settings. Second, this study ultimately included only 24 patients, and as an exploratory investigation, no formal sample size calculation was performed. The primary outcome, the longitudinal changes in SF-12 PCS at T1, T2, and T3, was analyzed using the Friedman test, which yielded a p-value of 0.07, indicating no statistically significant difference. However, this result may be influenced by a type II error due to the small sample size and therefore should be interpreted with caution. Third, attrition was substantial: 25 of the 49 enrolled participants (51%) were excluded, primarily because they were unable to return home or did not respond. This high dropout rate poses a significant threat to generalizability. Moreover, the excluded participants tended to be older and had longer care durations, suggesting potential selection bias. Forth, the use of paper-based self-administered questionnaires may have limited participation among individuals with functional impairments. While electronic PROs could improve response rates, they would require additional support for older users [27]. Despite these limitations, no significant differences in age, sex, or baseline ADL were observed between included and excluded participants, which partially mitigates concerns about attrition bias [28].

## Conclusions

This study demonstrates that older adults transitioning between home and the ROKEN experience significant HRQoL changes, particularly in social roles and overall social care-related quality of life. While mental health improved during facility admission, social engagement and perceived autonomy decreased. These findings underscore the importance of integrating PROs into care planning to optimize the long-term well-being of older adults.
